# Evolution of Antimicrobial Resistance in *Acinetobacter baumannii* and *Pseudomonas aeruginosa* in Romania: A Narrative Review of Published Data (2001–2024)

**DOI:** 10.3390/microorganisms14020429

**Published:** 2026-02-11

**Authors:** Elena Roxana Buzilă, Olivia Simona Dorneanu, Luminița Smaranda Iancu, Felicia Trofin, Alexandru Duhaniuc, Costin Damian, Cătălina Luncă

**Affiliations:** 1 Grigore T. Popa University of Medicine and Pharmacy Iasi, 700115 Iași, Romania; elena-roxana.buzila@umfiasi.ro (E.R.B.); olivia.dorneanu@umfiasi.ro (O.S.D.); felicia.trofin@umfiasi.ro (F.T.); alexandru.duhaniuc@umfiasi.ro (A.D.); costin-damian@umfiasi.ro (C.D.); catalina.lunca@umfiasi.ro (C.L.); 2 National Institute of Public Health, Iasi Regional Center for Public Health, 700465 Iasi, Romania; 3 Clinical Hospital of Infectious Diseases “Sf. Parascheva”, 700116 Iasi, Romania; 4 “Sf. Spiridon” Clinical Emergency Hospital, 700111 Iasi, Romania; 5 Emergency Clinical Hospital for Children “Sf. Maria”, 700309 Iasi, Romania

**Keywords:** antibiotic resistance evolution, *Acinetobacter baumannii*, *Pseudomonas aeruginosa*, dissemination of antibiotic-resistant strains, public health policies

## Abstract

Antimicrobial resistance constitutes a major global public health challenge, with Romania among the European countries affected. *Acinetobacter baumannii* and *Pseudomonas aeruginosa* was designated by the World Health Organization (WHO) as priority pathogens, are frequently implicated in healthcare-associated infections (HAIs), including pneumonia, surgical site infections, urinary tract infections, and bacteremia. We have narratively synthesized surveillance studies published in different regions of Romania, encompassing date whit the period 2001–2024. A total of 13 studies reporting data on *P*. *aeruginosa* (2003–2024) and 15 studies on *A*. *baumannii* (2001–2024) were included. The strains were isolated from various pathological products, including urine, sputum, tracheobronchial aspirate, blood, bronchoalveolar lavage, pus, peritoneal fluid, and cerebrospinal fluid, from hospitalized patients in different clinical departments. This review evaluated long-term trends in antimicrobial resistance profiles of *A*. *baumannii* and *P*. *aeruginosa* strains circulating in various regions of Romania. The findings revealed both temporal variability in resistance rates within comparable intervals and differences across distinct time periods. These long-term trends underscore the need for sustained national surveillance systems, harmonized reporting practices, and reinforced antimicrobial stewardship programs.

## 1. Introduction

Antimicrobial-resistant (AMR) bacteria are a leading cause of healthcare–associated infections (HAI) worldwide, and are increasingly appearing in community-acquired infections [1].

Governments around the world are increasingly recognizing the risk posed by AMR, considered a major threat to modern medicine [2,3].

Diseases caused by multidrug-resistant (MDR) organisms substantially increase the severity of illness, mortality rates, and overall healthcare costs [1]. Data from 2019 indicate that antibiotic resistance directly caused 1.27 million deaths and was associated with 4.95 million deaths overall [4,5]. Before the pandemic, coordinated efforts to control infectious diseases had contributed to a reduction in AMR-related deaths, especially in hospitals. However, the period that followed witnessed a troubling resurgence of nosocomial multidrug-resistant Gram-negative bacilli infections [6]. Cumulatively from 2025 to 2050, the reference scenario forecasts 39·million deaths attributable to AMR and 169 million deaths associated with AMR [7]. The so-called golden era of antibiotic discovery concluded in the 1980s, after which the emergence and widespread dissemination of antibiotic resistance became a significant challenge for scientific and public health institutions worldwide [8]. Consequently, the rising prevalence of antibiotic resistance has developed into a major global public health concern [9]. The spread of AMR, largely driven by inappropriate and excessive antibiotic use, has generated complex challenges affecting human, animal, and environmental health, imposing substantial societal and economic burdens [3].

Although the development of antibiotic resistance in microorganisms is a natural evolutionary process, it is markedly accelerated by the overuse and misuse of antimicrobial agents in both human and veterinary medicine [2,10]. Inappropriate antibiotic use includes administration in the absence of bacterial infection (e.g., for viral or non-infectious conditions), as well as improper antibiotic selection, dosing, or treatment duration [10]. Over the past three decades, such practices have exerted strong selective pressure favoring the emergence and dissemination of resistant bacterial strains. As a result, the treatment of bacterial infections increasingly requires higher antibiotic doses and prolonged therapies, leading to longer hospital stays and increased healthcare-associated costs [11].

In 2014, the World Health Organization (WHO) named antibiotic resistance as one of the three most important public health threats of the 21st century [12]. Subsequently, in 2017, the WHO designated *A*. *baumannii* as a priority pathogen for research and development of new antimicrobial therapies and ranked *P*. *aeruginosa*, alongside *A*. *baumannii* and *Enterobacterales*, among the top three pathogens requiring urgent drug development [13]. In 2018, *A*. *baumannii* was further included in the ESKAPE group of highly virulent, MDR pathogens, reinforcing its status as a critical priority for AMR research and therapeutic innovation [14].

In Romania, AMR data are reported to EARS-Net (European Antimicrobial Resistance Surveillance Network) primarily for isolates recovered from blood and cerebrospinal fluid, which are collected through a limited number of reference laboratories. EARS-Net does not provide clinical data, regional distribution, or patient information; EARS-Net is excellent for European comparisons, but insufficient for assessing the real national burden. Available surveillance data place Romania among the European countries reporting some of the highest levels of AMR. According to EARS-Net, data reported by Romania in 2023 showed an estimated population coverage of 13% and low geographic and hospital representativeness with a low number of reported isolates [15]. This approach represents a limitation, as the microorganisms analyzed are also involved in non-invasive infections, and the reported data are not sufficient for a complete assessment of resistance trends and for adequate guidance of antibiotic therapy.

The COVID-19 pandemic has significantly transformed medical practice worldwide, resulting in the suspension of routine care and the postponement of many surgical procedures. Hospitalizations surged, and the intensive care unit (ICU) became overwhelmed with critically ill patients [6,16]. The heightened use of antibiotics during this period contributed to rising AMR, particularly among non-fermenting Gram-negative bacteria such as *A*. *baumannii* and *P*. *aeruginosa*, complicating treatment and extending hospital stays [16]. Additionally, the pandemic saw a marked increase in *Acinetobacter*-related nosocomial infections, driven by prolonged ICU stays, mechanical ventilation, and greater reliance on oxygen therapy [6,16].

Motivation

Antibiotic resistance is an evolving phenomenon, influenced by a multitude of factors, including the inappropriate use of antimicrobials in human and veterinary medicine, horizontal gene transfer between bacterial populations, the effectiveness of infection prevention and control measures, and population mobility patterns. Therefore, long-term monitoring of resistance trends is essential to understand how these factors influence the emergence and dissemination of antibiotic-resistant strains in both community and hospital settings. Longitudinal datasets facilitate early detection of antibiotic-resistant antimicrobial agents and support the development of up-to-date, evidence-based therapeutic guidelines. Analysis of data collected over extended periods enhances the ability to assess epidemiological risk, prioritize public health interventions, and inform the development of local and national policies aimed at mitigating AMR.

Aim

The purpose of this narrative review is to synthesize and analyze existing data on the evolution of antibiotic resistance among strains of *A*. *baumannii* and *P*. *aeruginosa* circulating in the Romanian hospital’s environment. The paper aims to highlight the trends in AMR, the mechanisms involved, and the factors that favor the selection and spread of these pathogens. The article also aims to identify knowledge gaps and suggest future research directions and strategies for resistance control.

## 2. The Spectrum of Clinical Infections Caused by *Acinetobacter baumannii* and *Pseudomonas aeruginosa*

*A*. *baumannii* and *P*. *aeruginosa* are opportunistic pathogens responsible for a broad spectrum of clinical infections, being associated with conditions such as pneumonia, urinary tract infections, osteomyelitis, skin and soft tissue infections, bloodstream infections, meningitis, endocarditis, septicemia, as well as ocular and otic infections [17,18]. These microorganisms play a significant role in nosocomial pathology, being frequently involved in ventilator-associated pneumonia and hospital-acquired bloodstream infections [19,20].

A recent study also indicated that *A*. *baumannii*, while accounting for only 6% of all hospital-acquired infections, was responsible for 60% of pneumonia cases and 25% of bloodstream infections [20].

It is estimated that *P*. *aeruginosa* has a prevalence of 7.1–7.3% amongst all HAI. In ICU patients, *P*. *aeruginosa* is responsible for an even higher percentage of HAI. A large international observational point-prevalence study of infections in ICU patients found that *P*. *aeruginosa* represented 16% of patient infections and was the cause of 23% of all ICU-acquired infections, with a respiratory source being the most common site of *P*. *aeruginosa* infection [21]. *P aeruginosa* is also a major cause of morbidity and mortality among hospitalized burn patients globally [18,22].

In Romania, HAI and AMR constitute a significantly overlooked pathology. Official hospital reports indicate that HAI prevalence rates were only 0.2–0.25% in 2018. A European study conducted in 2018 identified more precise data for Romania, indicating a prevalence of 2.6%, which corresponds to approximately 100,000 cases registered annually in the country [23].

Both *P*. *aeruginosa* and *A*. *baumannii* possess a remarkable capacity to form biofilms, which confers them the ability to cause severe infections associated with significant morbidity and mortality. Biofilm formation facilitates the survival of these microorganisms on synthetic or abiotic surfaces, allows them to evade the host immune response, and confers increased resistance to antimicrobial therapies [24,25,26,27]. In the case of *A*. *baumannii*, this adaptation determines prolonged persistence in the hospital environment, even under unfavorable conditions that would be lethal to other Gram-negative bacteria, thus increasing the risk of HAI and favoring the emergence of nosocomial outbreaks [25,26,27]. Consequently, due to these characteristics, *A*. *baumannii* can be transmitted through direct contact with inert objects, being a constant threat to immunocompromised patients [25].

## 3. Intrinsic Resistance

### 3.1. Intrinsic Resistance for Pseudomonas aeruginosa

*P*. *aeruginosa* infections are becoming more difficult to treat because this bacterium is naturally resistant to many antibiotics, and the number of MDR and pandrug-resistant (PDR) strains is increasing worldwide. Strains that are resistant to almost all classes of commonly used antibiotics have been reported, including aminoglycosides, cephalosporins, fluoroquinolones, and carbapenems [28]. Wild-type strains of *P*. *aeruginosa* are resistant to amoxicillin (with or without clavulanate), first- and second-generation cephalosporins, cefotaxime, ceftriaxone, and ertapenem, while they remain susceptible to ticarcillin, piperacillin, ceftazidime, cefepime, imipenem, meropenem, and doripenem. Aztreonam activity is variable. Clavulanate is a strong inducer of AmpC in *P*. *aeruginosa*, and experimental data suggest a risk of clinical failure with the ticarcillin–clavulanate association [29].

*P*. *aeruginosa* possesses intrinsic resistance to most antibiotics through the presence of the inducible cephalosporinase AmpC, the efflux pumps MexAB-OprM and MexXY, low outer membrane permeability, and oxacillinases, all of which are chromosomally encoded [30,31].

The inducible cephalosporinase AmpC confers natural resistance to aminopenicillins (amoxicillin and ampicillin) and cephalosporins (cefoxitin, cefuroxime, and ceftriaxone) [31]. The activity of this enzyme is not affected by beta-lactamase inhibitors (e.g., clavulanic acid, sulbactam, and tazobactam) that are commonly used in clinical practice [19].

An important contribution to the natural antibiotic resistance of *P*. *aeruginosa* strains is made by efflux pumps. Twelve different RND (Resistance–Nodulation–Division) efflux systems have been described for this pathogen [30].

MexAB-OprM confers resistance to beta-lactamases (except imipenem), fluoroquinolones, trimethoprim, sulfonamides, chloramphenicol, and tetracyclines, while MexXY-OprM is involved in resistance to cefepime, aminoglycosides, fluoroquinolones, tetracyclines, chloramphenicol, trimethoprim, sulfonamides, and macrolides [32].

### 3.2. Intrinsic Resistance for Acinetobacter baumannii

*A*. *baumannii* is intrinsically resistant to several groups of antimicrobials, including glycopeptides, lincosamides, macrolides, and streptogamins [33].

The mechanisms underlying intrinsic resistance consist of the natural membrane impermeability, basal efflux activity, and the presence of two chromosomally encoded beta-lactamases: an ADC cephalosporinase and an OXA-51 oxacillinase [32].

To date, three efflux systems have been described for *A*. *baumannii*, which are encoded by different operons, *ade*ABC, *ade*FGH, and *ade*IJK, and belong to the RND family. The Ade IJK efflux system confers low-level resistance to β-lactams, tetracyclines, macrolides, lincosamides, phenicols, fusidic acid, and fluoroquinolones [32].

Virtually all *A*. *baumannii* isolates possess the gene encoding OXA-51 or one of its related variants that confer very low carbapenem resistance. The expression of this β-lactamase becomes increased when mobile elements like the insertion sequence ISAba1 are positioned upstream of this gene in an opposite orientation and act as promoter sequences [34].

Because the *bla*_OXA-51_ gene is usually located chromosomally, it is used as a marker to identify bacteria belonging to the species *A*. *baumannii* [35]. However, more and more studies have reported that the *bla*_OXA-51_ gene is also present in other *Acinetobacter* spp., indicating that detection of this gene is not reliable for identifying *A*. *baumannii* strains [36].

## 4. Factors Favoring the Emergence and Dissemination of Antibiotic-Resistant Strains

Antibiotic resistance predates the therapeutic use of antibiotics in humans, which began in the 1940s [37]. AMR is a major threat to global health because microorganisms (bacteria, viruses, fungi, and parasites) develop biological mechanisms that reduce the effectiveness of antimicrobial agents, mainly through genetic changes that allow them to survive in their presence [38].

Antibiotic pressure and other stressors can select bacteria that rapidly accumulate mutations that promote resistance. Spontaneous mutation is an essential mechanism of adaptation. In addition, mobile genetic elements—such as insertion sequences, integrons, transposons, and plasmids—facilitate the spread and accumulation of resistance genes in microbial populations [1,39].

Acquired resistance in *P*. *aeruginosa* occurs either through the horizontal transfer of resistance genes or through genetic mutations. Bacteria can obtain these genes via horizontal gene transfer from other strains of the same species or even from different bacterial species. Mutations may lead to the overexpression of antibiotic-inactivating enzymes. In addition, mutational changes can reduce antibiotic uptake, modify antibiotic target sites, and increase the expression of efflux pumps and inactivating enzymes—all of which enable the bacteria to survive in the presence of antimicrobial agents [30].

Similarly, *A*. *baumannii* isolates can develop resistance through several mechanisms, including enzymatic degradation of antibiotics, modification of the target site, altered membrane permeability, and the presence of multidrug efflux pumps [40].

Surveillance of antibiotic use provides a basis for improving antibiotic stewardship. Identifying trends in use can help tailor educational and vaccination campaigns, policy recommendations, and clinical guidelines to the unique challenges of each region or country [5].

Inadequate antibiotic dosing, incomplete treatment, and irrational use of antimicrobial courses are recognized as major drivers in the development and dissemination of AMR [41,42,43]. This mechanism is rooted in natural selection, whereby resistant strains survive and proliferate, while susceptible populations are eliminated [41,42]. Antibiotics have also been widely administered in dairy and livestock farming as a preventive measure, effectively functioning as group-level prophylaxis for food-producing animals [44]. However, Regulation European Union (EU) 2019/6, in force since 28 January 2022, restricts the prophylactic use of antibiotics in veterinary practice, permitting it only in exceptional circumstances and solely for individual animals [45].

According to the most recent data from 2021, Romania ranks first among EU Member States in the consumption of antibacterial for systemic use across both community and hospital settings, with more than 3% of the population using antibiotics on a daily basis [46,47].

The biofilm matrix plays a key role in promoting antibiotic resistance by providing mechanical and biochemical protection that diminishes the efficacy of antimicrobial agents. Under such conditions, conventional antibiotics struggle to eradicate embedded bacteria. Additionally, nutrient limitation within biofilms can induce a transient state of antibiotic tolerance, explaining why bacteria residing in deeper layers appear more resistant. Once removed from the biofilm and cultured in vitro, these cells typically regain susceptibility, indicating a phenotypic rather than genotypic form of resistance [48].

Biofilms readily form on medical devices and hospital equipment—such as prosthetic joints, ventilators, and urinary or intravascular catheters—facilitating pathogen entry into the host. The microorganisms may penetrate the skin or respiratory tract, making hospitalized and immunocompromised patients particularly susceptible to infection [40].

A study by Codru et al. shows that the persistent colonization and rising resistance of *A*. *baumannii*, *Klebsiella pneumoniae*, and *Staphylococcus aureus* indicate that ICU pathogens may rely on biofilm formation on medical devices for survival [49].

Human exposure to antibiotic-resistant pathogens occurs predominantly through contact with other individuals in both clinical and community environments, via direct or indirect transmission, aerosols, or contaminated food [50]. *P*. *aeruginosa* and *A*. *baumannii* may spread from contaminated surfaces—including bedding, curtains, bedrails, sinks, doors, feeding tubes, and medical equipment—with ventilators, suction catheters, and intravascular devices representing major infection sources [51]. Colonized patients function as reservoirs, and healthcare workers’ hands can disseminate pathogens between patients and surfaces [52]. In the ICU, gastrointestinal colonization with *A*. *baumannii* or *P*. *aeruginosa* is a prerequisite for the occurrence of clinical infections, hospital outbreaks, and widespread dissemination of these strains, which are most frequently multi-resistant to antibiotics [53,54].

The environment acts as a reservoir of antibiotic-resistant strains [55], and hands remain the most critical vector for transmission in healthcare environments [56]. Healthcare workers may acquire microorganisms through contact with patients or the clinical environment, and subsequently transfer them to other patients. Contaminated sinks, showers, respiratory equipment, or staff hands represent common routes of acquisition, with intensive care and immunocompromised patients at the highest risk of infection [52,56].

A major means of dissemination of antibiotic-resistant microorganisms and antibiotic resistance genes (ARG) into soil, water, and even air is hospital wastewater and agricultural runoff [55]. The uncontrolled disposal of antibiotic residues and other co-contaminants into the environment favors the selection of resistant bacteria, which subsequently develop, multiply, and spread in different environmental matrices, leading to increased abundance of ARGs [37].

Hospital effluents are considered hot spots for the presence and dissemination of antibiotic-resistant bacteria in the natural environment [57]. Wastewater treatment plants, although essential, can facilitate the survival, multiplication, and dissemination of these microorganisms; rivers are also major reservoirs, as they receive urban, hospital, industrial, and domestic wastewater. Thus, aquatic environments simultaneously function as reservoirs and routes of transmission for the dissemination of antibiotic-resistant bacteria, highlighting the critical need for effective waste management and treatment to limit the spread of AMR [55,58].

Numerous studies have reported the occurrence of carbapenem-resistant *A*. *baumannii* and *P*. *aeruginosa* in wastewater across various geographical regions worldwide, including Romania [59,60,61,62,63,64,65].

Rapid identification of high-risk clones is essential for isolating infected patients, preventing the spread of resistance, and improving antimicrobial treatment. This requires knowledge of the genetic environment and transport platforms of ARGs, as well as the development of new methods for assessing the potential spread of ARGs mediated by mobile genetic elements, both in aquatic and hospital environments [62].

## 5. The Evolution of Resistance

*A*. *baumannii* and *P*. *aeruginosa* are major hospital pathogens, recognized for their ability to rapidly develop resistance mechanisms to multiple antibiotics. The emergence of resistance to most of the antibiotics available to treat infections caused by *P*. *aeruginosa* and *A*. *baumannii* can become a challenge for doctors because a limited number of therapeutic options remain available [66].

Until the early 1970s, infections caused by *Acinetobacter* spp. were treated with carbenicillin, gentamicin, and nalidixic acid, either alone or in combination. After 1975, high rates of resistance were observed, and currently, ureidopenicillin, aminopenicillins, extended-spectrum cephalosporins, tetracycline, chloramphenicol, cephamycin such as cefoxitin, and most aminoglycosides are no longer effective against strains of *Acinetobacter* spp. [51].

The COVID-19 pandemic represented a major challenge for healthcare systems, not only through the management of viral infections, but also through the indirect impact on antimicrobial use and infection control. Hospital overcrowding and disruption of stewardship programs have led to an increase in empirical antibiotic prescribing, including in situations with a low probability of bacterial coinfection. In the early stages of the pandemic, broad-spectrum antibiotics, especially carbapenems, were frequently administered to patients with COVID-19, especially in the ICU. This practice, driven by diagnostic uncertainty, case severity, and the lack of rapid tests to differentiate viral from bacterial infections, has contributed to the excessive use of antibiotics [67].

A 2020 study by Andrews et al. found a decrease in total antibiotic use in hospitals compared to 2015–2019, along with an increase in the use of antibiotics for respiratory infections and broad-spectrum antibiotics [68].

Other research has confirmed this trend, reporting an increase in the use of broad-spectrum antibiotics, especially those classified as “Reserve” by the WHO, as well as third-generation cephalosporins and fluoroquinolones during the COVID-19 pandemic [69,70]. The excessive use of antibiotics both in healthcare facilities and in the community, in the context of the pandemic, may have contributed to the amplification of AMR [71].

In many situations, the prescription of antibiotics has been used as a measure to exclude bacterial infections that mimic COVID-19 symptoms, such as bacterial pneumonia, or as an adjunct in the management of complications associated with viral infection [72].

AMR monitoring is essential for guiding therapy and reducing the impact of healthcare-associated infections. According to the European Centre for Disease Prevention and Control (ECDC), when combined resistance exceeds the 50% threshold, therapeutic options become severely limited. The persistence of *Acinetobacter* spp. in the hospital environment amplifies their impact, as they are difficult to eradicate and promote continued transmission [73].

In Table 1 and Table 2 we have summarized the evolution of antibiotic resistance for *P*. *aeruginosa* and *A*. *baumannii* strains isolated in Romania, highlighting the changes in the percentages of resistance to different antibiotics in the pre-pandemic (2001–2019), pandemic (2020–2021), and post-pandemic (2023–2024) periods and different country geographical regions (East, West, South, Southeast, and Southwest). These data allow the assessment of epidemiological trends and the substantiation of control strategies and rational use of antibiotics.

This analysis was based on data collected in Romania between 2003 and 2024 for *P*. *aeruginosa* (13 studies) and 2001–2024 for *A*. *baumannii* (15 studies). The strains were isolated from various pathological products, including urine, sputum, tracheobronchial aspirate, blood, bronchoalveolar lavage, pus, peritoneal fluid, and cerebrospinal fluid, from patients hospitalized in different clinical departments. For antimicrobial susceptibility testing (AST), six studies used automated systems, eight studies applied the disk diffusion method, and five studies employed both approaches; in one study, the AST method was not specified. Regarding result interpretation, seven studies reported using EUCAST (*European Committee on Antimicrobial Susceptibility Testing*) standards [93], nine studies applied CLSI (*Clinical and Laboratory Standards Institute*) criteria [94], two studies used both standards, and in two studies, the interpretative standard was not reported. For the classification of resistance phenotypes, two studies used the definitions proposed by Magiorakos et al. [95], one study applied the criteria described by Falagas et al. [96], while in one study, the criteria used to classify resistance phenotypes were not specified.

Most studies were conducted in the Southern Region of the country and reported for *P*. *aeruginosa* the temporal changes in resistance rates, and rates of MDR strains ranging from 30 to 85% [78,82,84].

In studies that analyzed clinical isolates from different pathological products, *P*. *aeruginosa* was the most common pathogen identified from respiratory samples, rep-resenting 30% [76] and 11% [77] of the total isolates, respectively.

Studies conducted in the pre-pandemic period (2003–2018) [73,74,75,76] reported a wide range of resistance estimates for *P*. *aeruginosa*, with the highest rate reported for gentamicin in some studies (up to 72.58%), although estimates varied substantially. Antibiotic resistance rates of isolates from ICU and surgical wards were approximately twice as high as those from medical wards. Of the 529 isolates from adult hospitalized patients, 341 (64%) were identified from ICU/surgical wards [76].

In studies published after 2018 [72,78,79,80,81,82,83,84,85,86,87], resistance estimates for *P*. *aeruginosa* varied, and for amikacin, higher resistance was described compared with earlier periods [79], while resistance levels to carbapenems and ceftazidime remained relatively constant [78,79]. Studies conducted exclusively on strains isolated from urine [78,80,81] revealed lower rates of resistance compared to those from blood or the lower respiratory tract, where resistance to carbapenems and ceftazidime frequently exceeded 40–70% [75,82,84]. In Greece, for the periods 2013–2017 and 2020–2022, the resistance percentages for the same microorganism were below 40% [97].

A study focusing on *P. aeruginosa* strains isolated from the sputum of patients with community-acquired pneumonia reported relatively low levels of resistance to carbapenems (20%), while higher resistance levels were observed for ceftazidime and fluoroquinolones [83], which could be explained by their frequent use in the treatment of community-acquired infections and by the retention of carbapenems as reserve antibiotics.

Prior to the COVID-19 pandemic, the incidence of *P*. *aeruginosa* infections was increasing, a trend also reflected in the AMR profile. The implementation of more rigorous antibiotic therapy management strategies during the pandemic resulted in a temporary reduction in incidence and resistance rates [82]. Thus, for strains isolated from blood, a decrease in resistance to carbapenems (17%) and ceftazidime (33%) was observed in 2022, along with an increase in resistance to amikacin (40%), compared to the pre-pandemic period (2017), when the values were 65%, 50% and 20%, respectively. Subsequently, in the post-pandemic period (2023), a further increase in resistance to carbapenems and ceftazidime was observed, reaching 75% for imipenem, 80% for meropenem, and 50% for ceftazidime [82].

Similar results to those obtained in studies conducted in Romania were reported by Bennis et al., who described variations in ceftazidime resistance rates from 22.2% in the pre-pandemic period to 56.3% post-pandemic [98]. Also, a study conducted in Turkey showed significant increases in resistance to meropenem, Piperacillin–Tazobactam, and ceftazidime in the post-pandemic period, highlighting the worsening of the AMR crisis in the ICU [99].

Studies conducted in Romania during the pandemic period (2020–2022) variable resistance estimates for *P*. *aeruginosa* strains isolated from various pathological products [85,86,87]. In the case of strains isolated from urine, were initially associated with resistance levels exceeding 60% for several antibiotics, followed by a decline to below 40% towards the end of the pandemic [72,81].

Similar patterns were described in Turkey [100]. A study by Apetroaei et al., which included the pandemic (2020–2022) and post-pandemic (2023–2024) periods, reported decreases in resistance to meropenem and imipenem, from 63% and 58% in 2021 to 31% and 29% in 2024, respectively. Resistance to cefepime and ciprofloxacin also declined, while resistance to ceftazidime decreased more modestly (from 54% to 42%). Susceptibility to ceftazidime avibactam ranged from 14% in 2022 to 57% in 2024 [87].

A study conducted in Iran, between January 2020 and January 2022, on *P*. *aeruginosa* isolates from various clinical samples, revealed the highest resistance rates to imipenem (91.8%), meropenem (91.5%), and cefepime (87.1%) [101].

In contrast, studies conducted in Turkey and China reported higher resistance estimates for *P*. *aeruginosa* strains isolated during the pandemic compared to the pre-pandemic period, particularly for imipenem, colistin, amikacin, ciprofloxacin, and ceftazidime [16,102].

Two studies conducted in Hungary on strains isolated from urine samples report similar values for resistance percentages to several antibiotics for the period 2012–2023: ciprofloxacin (61–11%), imipenem (23–18%), meropenem (26–16%) [103], data that are similar to those reported in another study also conducted in Hungary in the period 2004–2015 [104].

In Bulgaria for the period 2010–2014, the resistance percentages were higher than those reported for the same period in our country, reaching 80% for ceftazidime and over 70% for carbapenems [105].

We created color-coded tables (Table 3, Table 4 and Table 5) to present the AMR patterns of *P*. *aeruginosa* strains reported in Romanian studies, so as to provide a descriptive picture of antibiotic resistance for this microorganism across the geographical regions included in the study and time periods. The visual summaries illustrate a marked heterogeneity of resistance estimates for *P*. *aeruginosa* across different regions and study periods. We observed that for the southern region, we have more studies identified compared to the other regions. Variability was observed both between regions and within the same region, especially for the southern region, which is represented by data extracted from multiple studies.

The data summarized in Table 2 indicate variations in antibiotic resistance among *A*. *baumannii* strains, depending on the time period, geographical region, and type of clinical specimen analyzed. In studies conducted between 2001 and 2003 [88] and 2003–2012 [74], high rates of resistance to fluoroquinolones (especially ciprofloxacin) and amino-glycosides (amikacin) were observed. Resistance to carbapenems, including imipenem and meropenem, was also frequently observed, with estimates varying across studies, highlighting methodological and population-level heterogeneity.

During 2008–2018, studies conducted in the eastern, southern, and western regions of Romania indicate high percentages of resistance for most classes of antibiotics used in testing. Similar to the results reported for *P*. *aeruginosa*, variations in resistance rates were reported in the first phase of the COVID-19 pandemic, not exceeding 55% for most antibiotics [85]. However, some studies describe important fluctuations, with transient reductions followed by abrupt increases, suggesting an instability of resistance profiles. High proportions of MDR *A*. *baumannii* strains have been reported, and in some studies, extensively drug-resistant (XDR) and, sporadically, PDR strains have also been identified, especially in the southern regions [78,82]. Antibiotic resistance rates have shown a similar trend in studies conducted in Iran and China [101,106].

A study in China showed that after the onset of the COVID-19 pandemic in January 2020, there was a modest and short-lived decrease in antibiotic resistance rates for *A*. *baumannii*. However, they were consistently higher in the ICU compared to other wards. These rates subsequently increased again, with the long-term trend indicating that high levels of resistance are maintained, except for the carbapenem resistance rate, which had a decreasing trend [106].

Studies focusing on *A*. *baumannii* strains isolated from blood culture data show that, in the pre-pandemic period, resistance to carbapenems was 78% for imipenem and 72% for meropenem; in 2019, during the pandemic, the percentages ranged from 92% to 100% and remained at 100% in 2024. Fluoroquinolone resistance percentages were also variable (91.14% for ciprofloxacin and 39.24% for levofloxacin), reaching 100% for in 2024 [82,91]. The study by Golli et al. reported colistin resistance rates ranging from 72 to 99% during the pandemic and post-pandemic. And post-pandemic, almost 90% of strains were MDR, 5.32% were PDR, and 92.30% were carbapenem-resistant [91].

Susceptibility testing for *A*. *baumannii* strains isolated from the sputum of patients with community-acquired pneumonia revealed resistance rates ranging from 71 to 88% for ciprofloxacin, meropenem, amikacin, and cefoperazone, while for tigecycline it was 33% and for colistin 0% [83].

A study conducted in the southern region of Romania between 2021 and 2024, including *A*. *baumannii* isolates from multiple clinical specimens, reported wide variability in resistance estimates, with values exceeding 80% for several antibiotics in 2021 and ranging between 10% and 76% in 2024 [87]. These findings cannot be generalized at the national level, as a separate study focusing on isolates collected in 2021 from the western region reported substantially lower resistance estimates, ranging from 13% to 57% [85]. Together, these observations highlight marked regional heterogeneity in reported resistance rates across Romania.

The susceptibility of *A*. *baumannii* to colistin also varied depending on the period analyzed and the type of specimen. For strains isolated from blood, studies reported resistance rates ranging from 0 to 6% for the period 2017–2019, followed by values reaching 90–100% in the period 2020–2024 [82]. In the case of strains isolated from urine, colistin resistance remained relatively constant, around 21%, in the period 2018–2022 [78]. For strains originating from various pathological products, resistance rates varied between 1% and 18% in the period 2021–2024 [87].

Several studies reported higher resistance estimates for *A*. *baumannii* during the COVID-19 pandemic compared with the pre-pandemic period, particularly for imipenem, amikacin, ciprofloxacin, trimethoprim–sulfamethoxazole and ceftazidime, while resistance to colistin appeared largely unchanged across studies [16,93]. Isolates recovered from blood cultures also frequently showed higher levels of resistance during the pandemic, including increased resistance to cefoperazone/sulbactam [102].

Also, studies from countries neighboring Romania report similarly high levels of AMR in *A*. *baumannii*, with resistance exceeding 80–90% to most antibiotic classes, including carbapenems, aminoglycosides, fluoroquinolones, and trimethoprim–sulfamethoxazole in Bulgaria (2010–2014 and 2019–2021) [105,107], Greece (2013–2022 and 2016–2021) [97,108], and Italy (2015–2019 and 2020–2023) [109], while colistin generally remained the most active agent despite increasing resistance rates over time. Two studies conducted in Greece report the broad predominance of MDR strains, representing approximately 97% of isolates during the period 2016–2021 [108], while a separate analysis covering the period 2016–2023 identified the XDR phenotype in 15–55% of isolates [110].

As in studies in our country, colistin generally remained the most active agent [107], colistin resistance showed substantial variability and a worrying trend of increase over time, reaching up to 60% in Greece [97,108,110].

Overall, the data summarized in Table 2 indicate persistently high levels of AMR among *A*. *baumannii* isolates, with regional and temporal variability.

We also created color-coded tables (Table 6, Table 7 and Table 8) for *A*. *baumannii* that summarize the resistance estimates for this microorganism across study regions and time periods. In this case, consistently high levels of resistance to multiple antibiotic classes are observed, and regional and temporal variability is not as evident as in the case of *P*. *aeruginosa* strains. For this microorganism, as for *P*. *aeruginosa*, we observe a more consistent representation of studies from the south (Table 6) of the country compared to the other regions (Table 7 and Table 8).

Differences in reported resistance levels across regions and time periods likely reflect heterogeneity in study inclusion criteria, including the healthcare settings and hospital departments involved, the types of clinical specimens analyzed, and local laboratory practices such as antimicrobial susceptibility testing methods and interpretative standards. In addition, variability in the circulating bacterial populations may contribute to these differences. Consequently, observed regional patterns should be interpreted with caution and should not be assumed to represent uniform national resistance trends.

Sustained or increased use of antibiotics can lead to the selection of bacteria that develop resistance to those classes of antimicrobials. Romania has consistently reported high levels of antibiotic use compared to other European countries.Between 2009 and 2011, antibiotic consumption in Romania showed wide variability, ranging from 10.2 to 31.6 defined daily doses (DDDs) per 1000 inhabitants per day [111]. During the subsequent period (2011–2018), total antibiotic consumption remained consistent, with reported values between 26.29 and 26.66 DDD per 1000 inhabitants per day [112]. According to the European Surveillance of Antimicrobial Consumption Network (ESAC-Net), Romania ranked second in Europe for systemic antibacterial consumption in 2015, after Greece, with consumption levels remaining largely unchanged between 2011 and 2015 around the value of 30 DDD per 1000 inhabitants per day. It should be noted that Romania reports antimicrobial consumption data only for general care, which precludes differentiation between community and hospital sectors [113].

More recent data indicate that, during 2019–2023, total consumption of systemic antibacterials, combining community and hospital sectors, ranged between 27.47 and 28.8 DDD per 1000 inhabitants per day, remaining substantially higher than the EU/EEA average of approximately 20.0 DDD [15]. Projections suggest that, if current trends persist, antibiotic consumption in Romania may decrease by 2030; however, it is still expected to remain above the projected EU/EEA average (31.3 vs. 23.2 DDD per 1000 inhabitants per day, respectively) [114].

These data differentiate between antibiotic use in intensive care units and outside of intensive care units and cannot be directly linked to the resistance estimates summarized in this analysis. Therefore, they provide general context rather than evidence of a causal relationship between use and resistance.

## 6. Public Health Policies

AMR affects humans, animals, plants, and the environment, and MDR strains can be transmitted between species, including through direct contact or consumption of animal products. Managing AMR is beyond the capacity of any single sector and requires multisectoral coordination—across health, agriculture, industry, education, and the non-governmental sector—through both horizontal (across sectors) and vertical (national, regional, and international) collaboration [115].

The plans should include key elements, such as enhanced surveillance and strengthened infection prevention and control programs in hospitals and other healthcare settings, integrated with antimicrobial stewardship programs and good diagnostic practices [15].

Since 2014, the Joint Programming Initiative on Antimicrobial Resistance (JPIAMR) has launched transnational research calls, such as Strategic Research and Innovation. The JPIAMR brings together 27 countries to coordinate research and funding dedicated to AMR in a One Health approach. Through international collaboration, the initiative supports the development of antibacterial solutions, improved surveillance and diagnostics, and effective antibiotic use strategies. Agenda (2019–2024) sets global priorities: optimizing the use of antibiotics and therapeutic alternatives, improving diagnostics and surveillance, preventing the transmission of resistance, assessing the role of the environment, and strengthening infection control measures in the spirit of One Health [116].

In 2015, WHO launched the Global Antimicrobial Resistance and Utilization Surveillance System (GLASS) to strengthen resistance monitoring and inform strategies to combat it. Initially focused on bacteria involved in common human infections, the system has expanded to include antimicrobial consumption, invasive fungal infections, and a One Health framework. GLASS is continuously evolving to improve data quality and representativeness, and by the end of 2022, 127 countries and territories were enrolled [117].

In September 2024, the updated version of the European Partnership on Antimicrobial Resistance (EUP OHAMR) was published, a 10-year program 2025–2035, proposed by the European Commission under the Horizon Europe 2023/2024 work program. This program brings together 53 organizations from 30 EU and non-EU countries, providing joint support to research and innovation and mobilizing to address the challenges posed by AMR through a One Health approach [118].

Institutions such as the International Monetary Fund (IMF), World Bank, WHO, and G8 consider AMR a major threat to global health [119]. Underlining the need to address AMR requires a multidisciplinary and multisectoral One Health strategy that stimulates research and innovation to improve surveillance, diagnosis, and treatment, as well as to develop preventive measures aimed at reducing the use of antimicrobials and the spread of resistance [118,119,120].

Another important step is the study carried out through the ECDC’s European Antimicrobial Resistance Gene Surveillance Network (EURGen-Net), which will take place between October 2024 and June 2025, which will target carbapenem-resistant *A*. *baumannii* (CRAb) [121]. CRAb is classified by WHO as a critical priority pathogen because infections are difficult to treat due to widespread resistance to carbapenems and numerous other antimicrobials, leaving limited therapeutic options. According to ECDC, in 2019, almost 60,000 infections and over 2700 deaths directly attributable to CRAb were reported in hospitals in the EU/EEA [121].

In the absence of stronger and faster public health action, the EU will reach all its AMR targets by 2030. The consequence will be an increased number of infections with antibiotic-resistant bacteria that will be more difficult to treat, leading to increasing therapy challenges for patients and AMR-related deaths [15].

## 7. Conclusions

The study provides a national perspective on the evolution of antibiotic resistance in *A*. *baumannii* and *P*. *aeruginosa* strains circulating in Romania. Although the available data are limited and do not fully reflect the extent of the phenomenon, they provide valuable information that can guide the choice of appropriate therapeutic options. The results highlight the need to implement specific measures to identify the resistance profiles of the studied microorganisms and to conduct more extensive research, including both community and hospital-acquired strains. This review is an attempt to fill the gaps in the Romanian context and highlights the need for future studies to investigate the social and economic factors that influence antibiotic use, as well as other contextual factors that contribute to the discrepancies between knowledge and practice. Given the heterogeneity of antimicrobial susceptibility testing standards and methods, quantitative clustering and statistical trend analyses were not performed. The reported findings instead highlight substantial heterogeneity in resistance estimates, influenced by differences in the medical units analyzed, study periods, and methodological frameworks.

While EARS-Net offers standardized European surveillance of invasive infections, it does not capture the breadth of Romanian clinical data, including ICU-focused studies, non-invasive infections, and outbreak reports.

Environmental reservoirs, including hospital surfaces, water systems, and equipment, represent important potential sources of *P*. *aeruginosa* and *A*. *baumannii* dissemination. Future surveillance and research efforts should prioritize characterization of these reservoirs and the pathways by which resistant strains spread, as this could inform infection prevention, control strategies, and antimicrobial stewardship programs.

## 8. Limitations

This analysis has several limitations inherent to its narrative design. The reported resistance estimates were derived from studies that used different methods, standards, and AST breakpoints, which have evolved over time and limit direct comparability. Medical facilities varied, resulting in variations in pathology products, variations in patient categories, and these also contributed to heterogeneity. Interpretation of temporal changes was further complicated by revisions to EUCAST and CLSI breakpoints during the study period, which are documented in successive guideline updates. Finally, although temporal coincidences with events such as the COVID-19 pandemic, the descriptive nature of the data precludes causal inference.

## 9. Future Directions

AMR is a major global priority, especially for our country, and future research directions should focus on deepening the mechanisms involved in the dissemination of resistant strains and in developing control strategies for this phenomenon. Comparative studies carried out over long periods are needed, allowing the evaluation of the evolution of resistance to the main classes of antibiotics—carbapenems, aminoglycosides, fluoroquinolones, and colistin—among *A*. *baumannii* and *P*. *aeruginosa* strains isolated from hospitals in Romania.

Also, conducting distinct studies for each type of pathology would facilitate the development of diagnostic and treatment guidelines better adapted to the clinical context.

Investigation of emerging resistance mechanisms—including the production of metallo-β-lactamases, activation of efflux systems, and the occurrence of mutations in antibiotic targets—is crucial for understanding the dynamics of AMR. Comparing the genetic profiles of clinical strains with those isolated from the environment (wastewater) could provide valuable information on the circulation and persistence of resistance.

In addition, future studies should correlate antibiotic consumption at national and local levels with the evolution of resistant strains.

## Figures and Tables

**Table 1:** Evolution of antibiotic resistance for *Pseudomonas aeruginosa* strains circulating in Romania.

Year	Region	Total No	Pathological Product	ATS Determination Method/Interpretation Guide	Antibiotics													Reference
					CAZ %	IMI %	MEM %	AK %	G %	TOB %	CIP %	LEV %	TZP %	CT %	MDR %	XDR %	PDR %	
2003–2012	East	167	urine, sputum, pus, blood	disk diffusion method/CLSI	NA	46	NA	46	NA	NA	62	49	NA	0	NA	NA	NA	[74]
2008–2018	East	672	urine, sputum, tracheobronchial aspirate, pus, blood	disk diffusion method/CLSI, EUCAST	53.38	63.14	64.09	41.10	52.27	59.71	65.46	66.67	33.32	2.91	36	23.7	0.4	[75]
2010–2015	South	1296	sputum, bronchial aspirate, bronchial lavage	disk diffusion method/EUCAST	33	43	33	21	36	31	36.56	35.8	23.8	3.41	NA	NA	NA	[76]
2012–2013	West	62	wound exudate, urine, broncho-alveolar lavage, blood, cerebrospinal fluid, peritoneal fluid, central venous catheter, bile, ascites aspirate, pleural aspirate, vaginal discharge	disk diffusion method/EUCAST	51.61	48.39	48.39	45.16	72.58	NA	59.68	NA	54.84	NA	NA	NA	NA	[77]
2018–2019	South	255	Urine	automatic systems/EUCAST	46	43–41	43–44	36–24	NA	NA	NA	NA	NA	3–6	37	NA	NA	[78]
2018–2019	West	105	tracheobronchial secretion, wound discharge, blood, urine	automatic systems, disk diffusion method, EUCAST	54.28	47.61	47.61	56.19	56.19	NA	56.19	NA	6.66	8.57	14.28	1.90	39.04	[79]
2018–2019	South	57	urine	disk diffusion method/CLSI	24.56	16.0	16.9	14.03	NA	NA	NA	31.57	NA	NA	NA	NA	NA	[80]
2018–2019	South	33	urine	disk diffusion method/CLSI, EUCAST	27.27	30.3	0	15.15	NA	NA	NA	18.18	NA	NA	NA	NA	NA	[72]
2018	South	14	urine	disk diffusion method/CLSI	21.42	14.28	14.28	14.28	NA	NA	NA	21.42	NA	NA	NA	NA	NA	[81]
2017–2019	South	54	blood	automatic systems/EUCAST	65–36	50–46	45–43	20–15	NA	40–31	50–58	43–70	83–50	0	30–85	NA	NA	[82]
2017–2018	South	-	sputum	disk diffusion method/CLSI	75	NA	20	NA	NA	NA	75	NA	0	0	NA	NA	NA	[83]
2016–2020	South -East	366	urine, skin secretions/collections, soft tissues, blood	disk diffusion method, automatic systems/CLSI	66.5	NA	55.2	NA	61.6	NA	64.8	NA	73.7	NA	29	NA	NA	[84]
2020–2022	South	31	blood	automatic systems/EUCAST	13–33	37–17	37–17	29–40	NA	0–43	42–0	0–25	40–0	0–17	47–17	NA	NA	[82]
2021	West	286	blood, wound secretions, peritoneal fluid, genital secretions, urine, sputum, bronchial aspirate, catheter tip, pus	disk diffusion method, automatic systems/CLSI	14.6	12.9	8.3	8.6	14.9	NA	17.2	14.6	6.6	1.7	NA	NA	NA	[85]
2020–2021	South	31	urine	disk diffusion method/CLSI, EUCAST	67.74	77.41	9.67	51.61	NA	NA	NA	64.51	NA	NA	NA	NA	NA	[72]
2020	South	15	urine	disk diffusion method/CLSI	73.33	73.33	66.66	46.66	NA	NA	NA	93.33	NA	NA	NA	NA	NA	[81]
2022	South	13	urine	disk diffusion method/CLSI	38.46	30.76	30.76	30.76	NA	NA	NA	46.15	NA	NA	NA	NA	NA	[81]
2022	South	144	urine, sputum, blood, pus, central venous catheter	automatic systems/CLSI	25	NA	24.3	21.52	33.33	28.05	18.75	17.36	10.41	0	NA	NA	NA	[86]
2020–2022	South	255	urine	automatic systems/EUCAST	46–43	54–35	54–42	32–42	NA	NA	NA	NA	NA	4–8	NA	NA	NA	[78]
2021	South	460	bronchial secretions, sputum, urine and blood culture, peritoneal fluid, cerebrospinal fluid	NA/EUCAST	54	58	63	40	48	NA	57	52	52	8	NA	NA	NA	[87]
2022	South	460	bronchial secretions, sputum, urine and blood culture, peritoneal fluid, cerebrospinal fluid	NA/EUCAST	51	49	61	30	42	NA	49	51	48	4	NA	NA	NA	[87]
2023	South	460	bronchial secretions, sputum, urine and blood culture, peritoneal fluid, cerebrospinal fluid	NA/EUCAST	45	35	41	22	26	NA	31	39	43	2	NA	NA	NA	[87]
2024	South	460	bronchial secretions, sputum, urine and blood culture, peritoneal fluid, cerebrospinal fluid	NA/EUCAST	42	29	31	21	18	NA	28	27	22	10	NA	NA	NA	[87]
2023–2024	South	26	blood	automatic systems/EUCAST	50–42	75–42	80–42	22–8	NA	25–33	21–33	67–57	100–50	25–17	21	NA	NA	[82]

Year—study interval; Region—geographical region from which the strains were analyzed; Total No—number of strains included in the study; Pathological Product—pathological products from which the strains included in the studies were isolated; EUCAST—European Committee on Antimicrobial Susceptibility Testing; CLSI—Clinical and Laboratory Standards Institute, NA—Not available; CAZ—ceftazidime; IMI—imipenem; MEM—meropenem; AK—amikacin; G—gentamicin; TOB—tobramycin; CIP—ciprofloxacin; LEV—levofloxacin; TZP—Piperacillin–Tazobactam; CT—colistin; MDR—multidrug-resistant; XDR—extensively drug-resistant; PDR—pandrug-resistant.

**Table 2:** Evolution of antibiotic resistance for *Acinetobacter baumannii* strains circulating in Romania.

Year	Region	Total No.	Specimen	ATS Determination Method/Interpretation Guide	Antibiotics													Reference
					AK %	G %	TOB %	CIP %	LEV %	IMI %	MEM %	TZP %	SXT %	CT %	MDR %	XDR %	PDR %	
2001–2003	-	633	tracheal secretions, blood, urine, wound secretions	automatic systems/NA	84	NA	NA	93	NA	22	NA	NA	NA	NA	NA	NA	NA	[88]
2003–2012	East	139	urine, sputum, pus, blood	disk diffusion method/CLSI	94	NA	NA	96	88	53	NA	NA	NA	0	NA	NA	NA	[74]
2008–2010	South	147	cerebrospinal fluid, blood, urine, sputum, wood secretion, tracheobronchial aspirate	automatic systems, disk diffusion method/NA	18.86	NA	44.45	10.89	NA	19.18	19.18	NA	13.77	0.1	NA	NA	NA	[89]
2008–2018	East	322	urine, sputum, tracheobronchial aspirate, pus, blood	disk diffusion method/CLSI, EUCAST	69.35	79.91	43.23	83.33	70.24	73.76	80.15	81.32	72.33	3.33	37	48.8	NA	[75]
2010–2015	South	503	sputum, bronchial aspirate, bronchial lavage	disk diffusion method/EUCAST	63.77	72.73	49.75	81.72	76.1	77.28	69.88	78.12	NA	1.62	NA	NA	NA	[76]
2011–2015	West	37	bronchial secretions, blood, urine, venous catheter	automatic systems, disk diffusion method/CLSI	92	95	92	NA	NA	95	86	NA	NA	3	NA	NA	NA	[90]
2012–2013	West	161	wound exudate, urine, broncho-alveolar lavage, blood, cerebrospinal fluid, peritoneal fluid, central venous catheter, bile, ascites aspirate, pleural aspirate, vaginal discharge	disk diffusion method/EUCAST	77.64	87.58	NA	91.30	NA	93.17	93.17	95.03	NA	0	NA	NA	NA	[77]
2017–2018	South	-	sputum	disk diffusion method/CLSI	86	NA	NA	88	NA	NA	86	NA	NA	0	NA	NA	NA	[83]
2017–2019	South	47	blood	automatic systems/EUCAST	62–56	85–67	77–39	92–72	62	92–78	92–72	NA	85–72	0–6	92–72	NA	NA	[82]
2018–2019	South	38	urine	automatic systems/EUCAST	62–100	62–100	NA	NA	NA	62–86	62–86	NA	NA	21	73	NA	NA	[78]
2016–2020	South -East	28	urine, secretions/collections, skin and soft tissue, blood	disk diffusion method, automatic systems/CLSI	NA	55.6	NA	47.4	NA	NA	57.1	66.7	NA	NA	36,8	NA	NA	[84]
2021	West	123	blood, wound secretions, peritoneal fluid, genital secretions, urine, sputum, bronchial aspirate, catheter tip, pus	disk diffusion method, automatic systems/CLSI	23.4	53.9	NA	56.2	22.7	48.4	52.3	35.2	12.5	1.6	NA	NA	NA	[85]
2020–2022	South	95	blood	automatic systems/EUCAST	44–93	88–93	63–93	97–93	9–64	94–93	94–100	NA	72–93	0–7	90	NA	NA	[82]
2020–2022	South	-	urine	automatic systems/EUCAST	29–100	86–100	NA	NA	NA	100–100	100–83	NA	NA	21	NA	29–100	86–100	[78]
2020–2021	South -West	37	blood	automatic systems/CLSI	NA	95	NA	100	97	92	92	97	NA	72	NA	NA	NA	[91]
2021	South	279	bronchial secretions, sputum, urine, blood, peritoneal fluid, cerebrospinal fluid	NA/EUCAST	81	82	NA	90	90	90	90	91	83	18	NA	NA	NA	[87]
2022	South	279	bronchial secretions, sputum, urine, blood, peritoneal fluid, cerebrospinal fluid	NA/EUCAST	72	80	NA	80	79	80	80	82	79	5	NA	NA	NA	[87]
2022–2023	South -West	132	blood	automatic systems/CLSI	NA	92	NA	97	97	95	95	97	NA	99	90	5.32	NA	[91]
2023	South	279	bronchial secretions, sputum, urine, blood, peritoneal fluid, cerebrospinal fluid	NA/EUCAST	39	35	NA	40	40	32	42	42	38	1	NA	NA	NA	[87]
2023–2024	South	16	blood	automatic systems/EUCAST	80–100	60–100	60–100	90–100	50–100	100	90–100	NA	80–100	0	100	0	0	[82]
2023–2024	West	118	bronchial secretions, sputum, blood,	automatic systems/EUCAST	88.88	88.88	88.88	95	NA	95	95	NA	95	3.38	0.84	91.52	3.38	[92]
2024	South	279	bronchial secretions, sputum, urine, blood, peritoneal fluid, cerebrospinal fluid	NA/EUCAST	40	39	NA	66	68	48	76	73	10	18	NA	NA	NA	[87]

Year—study interval; Region—geographical region from which the strains were analyzed; Total No—number of strains included in the study; Pathological Product—pathological products from which the strains included in the studies were isolated; EUCAST—European Committee on Antimicrobial Susceptibility Testing; CLSI—Clinical and Laboratory Standards Institute; NA—Not available; AK—amikacin; G—gentamicin; TOB—tobramycin; CIP—ciprofloxacin; LEV—levofloxacin; IMI—imipenem; MEM—meropenem; TZP—Piperacillin–Tazobactam; SXT—trimethoprim-sulfamethoxazole; CT—colistin; MDR—multidrug-resistant; XDR—extensively drug-resistant; PDR—pandrug-resistant.

**Table 3:** Antibiotic resistance for *Pseudomonas aeruginosa* strains in southern region of Romania.

Year	Pathological Product	Antibiotics										Reference
		CAZ %	IMI %	MEM %	AK %	G %	TOB %	CIP %	LEV %	TZP %	CT %	
2010–2015	sputum, bronchial aspirate, bronchial lavage											[76]
2018–2019	urine											[78]
2018–2019	urine											[80]
2018–2019	urine											[72]
2018	urine											[81]
2017–2019	blood											[82]
2017–2018	sputum											[83]
2020–2022	blood											[82]
2020–2021	urine											[72]
2020	urine											[81]
2022	urine											[81]
2022	urine, sputum, blood, pus, central venous catheter											[86]
2020–2022	urine											[78]
2021	bronchial secretions, sputum, urine and blood culture, peritoneal fluid, cerebrospinal fluid											[87]
2022	bronchial secretions, sputum, urine and blood culture, peritoneal fluid, cerebrospinal fluid											[87]
2023	bronchial secretions, sputum, urine and blood culture, peritoneal fluid, cerebrospinal fluid											[87]
2024	bronchial secretions, sputum, urine and blood culture, peritoneal fluid, cerebrospinal fluid											[87]
2023–2024	blood											[82]

Year—study interval; Pathological Product—pathological products from which the strains included in the studies were isolated; CAZ—ceftazidime; IMI—imipenem; MEM—meropenem; AK—amikacin; G—gentamicin; TOB—tobramycin; CIP—ciprofloxacin; LEV—levofloxacin; TZP—Piperacillin–Tazobactam; CT—colistin; Brown—<1%; Light Blue—1% to <5%; Blue—5% to <10%; Green—10% to <25%; Yellow—25% to <50%; Red—>50%; Grey—no data reported.

**Table 4:** Antibiotic resistance for *Pseudomonas aeruginosa* strains in eastern and southeastern regions of Romania.

Year	Pathological Product	Antibiotics										Reference
		CAZ %	IMI %	MEM %	AK %	G %	TOB %	CIP %	LEV %	TZP %	CT %	
2003–2012	urine, sputum, pus, blood											[74]
2008–2018	urine, sputum, tracheobronchial aspirate, pus, blood											[75]
2016–2020	urine, skin secretions/collections, soft tissues, blood											[84]

Year—study interval; Pathological Product—pathological products from which the strains included in the studies were isolated; CAZ—ceftazidime; IMI—imipenem; MEM—meropenem; AK—amikacin; G—gentamicin; TOB—tobramycin; CIP—ciprofloxacin; LEV—levofloxacin; TZP—Piperacillin–Tazobactam; CT—colistin; Brown—<1%; Light blue—1% to <5%; Blue—5% to <10%; Green—10% to <25%; Yellow—25% to <50%; Red—>50%; Grey—no data reported.

**Table 5:** Antibiotic resistance for *Pseudomonas aeruginosa* strains in western region of Romania.

Year	Pathological Product	Antibiotics										Reference
		CAZ %	IMI %	MEM %	AK %	G %	TOB %	CIP %	LEV %	TZP %	CT %	
2012–2013	wound exudate, urine, broncho-alveolar lavage, blood, cerebrospinal fluid, peritoneal fluid, central venous catheter, bile, ascites aspirate, pleural aspirate, vaginal discharge										-	[77]
2018–2019	tracheobronchial secretion, wound discharge, blood, urine											[79]
2021	blood, wound secretions, peritoneal fluid, genital secretions, urine, sputum, bronchial aspirate, catheter tip, pus											[85]

Year—study interval; Pathological Product—pathological products from which the strains included in the studies were isolated; CAZ—ceftazidime; IMI—imipenem; MEM—meropenem; AK—amikacin; G—gentamicin; TOB—tobramycin; CIP—ciprofloxacin; LEV—levofloxacin; TZP—Piperacillin–Tazobactam; CT—colistin; Brown—<1%; Light Blue—1% to <5%; Blue—5% to <10%; Green—10% to <25%; Yellow—25% to <50%; Red—>50%; Grey—no data reported.

**Table 6:** Antibiotic resistance for *Acinetobacter baumannii* strains in southern region of Romania.

Year	Specimen	Antibiotics										Reference
		AK %	G %	TOB %	CIP %	LEV %	IMI %	MEM %	TZP %	SXT %	CT %	
2008–2010	cerebrospinal fluid, blood, urine, sputum, wood secretion, tracheobronchial aspirate											[89]
2010–2015	sputum, bronchial aspirate, bronchial lavage											[76]
2017–2018	sputum											[83]
2017–2019	blood											[82]
2018–2019	urine											[78]
2020–2022	blood											[82]
2020–2022	urine											[78]
2021	bronchial secretions, sputum, urine, blood, peritoneal fluid, cerebrospinal fluid											[87]
2022	bronchial secretions, sputum, urine, blood, peritoneal fluid, cerebrospinal fluid											[87]
2023	bronchial secretions, sputum, urine, blood, peritoneal fluid, cerebrospinal fluid											[87]
2023–2024	blood											[82]
2024	bronchial secretions, sputum, urine, blood, peritoneal fluid, cerebrospinal fluid											[87]

Year—study interval; Pathological Product—pathological products from which the strains included in the studies were isolated; AK—amikacin; G—gentamicin; TOB—tobramycin; CIP—ciprofloxacin; LEV—levofloxacin; IMI—imipenem; MEM—meropenem; TZP—Piperacillin–Tazobactam; SXT—trimethoprim-sulfamethoxazole; CT—colistin; Brown Cream—<1%; Light Blue—1% to <5%; Blue—5% to <10%; Green—10% to <25%; Yellow—25% to <50%; Red—>50%; Gray—no data reported.

**Table 7:** Antibiotic resistance for *Acinetobacter baumannii* strains in eastern and southeastern regions of Romania.

Year	Specimen	Antibiotics										Reference
		AK %	G %	TOB %	CIP %	LEV %	IMI %	MEM %	TZP %	SXT %	CT %	
2003–2012	urine, sputum, pus, blood											[74]
2008–2018	urine, sputum, tracheobronchial aspirate, pus, blood											[75]
2016–2020	urine, secretions/collections, skin and soft tissue, blood											[84]

Year—study interval; Pathological Product—pathological products from which the strains included in the studies were isolated; AK—amikacin; G—gentamicin; TOB—tobramycin; CIP—ciprofloxacin; LEV—levofloxacin; IMI—imipenem; MEM—meropenem; TZP—Piperacillin–Tazobactam; SXT—trimethoprim-sulfamethoxazole; CT—colistin; Brown Cream—<1%; Light Blue—1% to <5%; Blue—5% to <10%; Green—10% to <25%; Yellow—25% to <50%; Red—>50%; Grey—no data reported.

**Table 8:** Antibiotic resistance for *Acinetobacter baumannii* strains in western and southeastern regions of Romania.

Year	Specimen	Antibiotics										Reference
		AK %	G %	TOB %	CIP %	LEV %	IMI %	MEM %	TZP %	SXT %	CT %	
2011–2015	bronchial secretions, blood, urine, venous catheter											[90]
2012–2013	wound exudate, urine, broncho-alveolar lavage, blood, cerebrospinal fluid, peritoneal fluid, central venous catheter, bile, ascites aspirate, pleural aspirate, vaginal discharge											[77]
2021	blood, wound secretions, peritoneal fluid, genital secretions, urine, sputum, bronchial aspirate, catheter tip, pus											[85]
2020–2021	blood											[91]
2022–2023	blood											[91]

Year—study interval; Pathological Product—pathological products from which the strains included in the studies were isolated; AK—amikacin; G—gentamicin; TOB—tobramycin; CIP—ciprofloxacin; LEV—levofloxacin; IMI—imipenem; MEM—meropenem; TZP—Piperacillin–Tazobactam; SXT—trimethoprim-sulfamethoxazole; CT—colistin; Brown —<1%; Light Blue—1% to <5%; Blue—5% to <10%; Green—10% to <25%; Yellow—25% to <50%; Red—>50%; Grey—no data reported.

## Data Availability

The original contributions presented in this study are included in the article. Emergency Clinical Hospital for Children “Sf. Maria”, 700309 Iasi, Romania. Further inquiries can be directed to the corresponding author.

## Abbreviations

The following abbreviations are used in this manuscript:

AMR	Antimicrobial Resistance
HAIs	Healthcare-Associated Infections
WHO	World Health Organization
EARS-Net	European Antimicrobial Resistance Surveillance Network
ICU	Intensive Care Unit
RND	Resistance–Nodulation–Division
EU	European Union
ARGs	Antibiotic Resistance Genes
CAZ	Ceftazidime
IMI	Imipenem
MEM	Meropenem
AK	Amikacin
G	Gentamicin
TOB	Tobramycin
CIP	Ciprofloxacin
LEV	Levofloxacin
TZP	Piperacillin–Tazobactam
CT	Colistin
MDR	Multidrug-Resistant
XDR	Extensively Drug-Resistant
PDR	Pandrug-Resistant
SXT	Trimethoprim–Sulfamethoxazole
ECDC	European Centre for Disease Prevention and Control
EEA	European Economic Area
GLASS	Global Antimicrobial Resistance and Utilization Surveillance System
JPIAMR	Joint Programming Initiative on Antimicrobial Resistance
EUP OHAMR	European Partnership on Antimicrobial Resistance
EURGen-Net	European Antimicrobial Resistance Gene Surveillance Network
CRAb	Carbapenem-Resistant *A*. *baumannii*
IMF	International Monetary Fund
ESAC-Net	European Surveillance of Antimicrobial Consumption Network
EUCAST	European Committee on Antimicrobial Susceptibility Testing
CLSI	Clinical and Laboratory Standards Institute
